# Illinois State Dental Society

**Published:** 1888-08

**Authors:** 


### Illinois State Dental Society.

(REPORTED FOR DENTAL REGISTER BY MRS. M. W. J.)

The Illinois State Dental Society convened in twenty-fourth annual session at Cairo, May 8, 1888.

OFFICERS.

PRESIDENT—C. B. ROHLAND, Alton.
VICE-PRESIDENT—CHARLES HENRY, Jacksonville.
SECRETARY—GARRETT NEWKIRK, Chicago.
TREASURER—T. W. PRICHETT, Whitehall.
LIBRARIAN—W. B. AMES, Chicago.

The meeting was called to order by the President at 10 A. M., and opened with prayer. The roll-call showed fifty-four members present. A hearty welcome was tendered the society on the part of the Mayor and citizens of Cairo. The response of Dr. C. R. E. Koch was eloquent and full of wit and humor.

The annual report of the Secretary showed an active membership of 142. The report of the Treasurer showed the receipt of over $700 during the year. The report of the Librarian showed no addition to either library or museum.

The morning session closed with the annual address of the President. His special topic was the ignorance of the masses in matters pertaining to dentistry and the importance of popular education on this subject. He spoke of this as the great bar to advancement notwithstanding the great strides made in other directions. The dentist fails signally in his duty who only fills teeth or makes substitutes. If a man falls in a pit, he must not only be lifted out, but taught how to keep out. Patients must not only have their teeth filled, but must be taught how to prevent recurrence of decay; to this end there should be lectures in the public schools, articles in the daily journals, lessons in the kindergardens, text-books on dental hygiene, anatomy and physiology, etc. He spoke of the action taken by the American Association, a committee having been appointed to report on this subject at Louisville, in August, before the joint meeting of the American and Southern Associations. He spoke of the action taken by the American Medical Association, in the formal recognition of Dentistry as a branch of Medicine, and of the proper attitude to be preserved by the dental profession—the proffered hand should be kindly taken and the snubs of the past overlooked—recognizing the new obligations imposed by the recognition, especially the necessity for raising the standard of dental education.

He spoke of the International Medical Congress and the work accomplished by the dental section, of the steps taken by the various societies to resist the "Bridge and Crown Patents," and of the importance that all should work together in the matter, remembering the days of Josiah Bacon. He referred to the coming twenty-fifth annual meeting of the society and the propriety of celebrating it in a suitable manner; also to the rightful claim of the members of the dental profession to exemption from jury duty.

A proposed amendment to the constitution of the society raised a discussion of the time requisite for graduation, and the adoption of a resolution, introduced by Dr. Harlan, urging upon the National College of Dental Faculties to extend the time to three years, including two full courses at college, in separate years. The report of the committee on Dental Science and Literature, referred with satisfaction to the progress made in eliminating antique modes of practice, in the growing freedom from empiricism, in the permanent salvation of countless teeth under conditions formerly condemned to the forceps, especially through the operation of crowning. More conservative practice in preserving live pulps by capping was urged, in view of the hobby for immediate root filling now attracting so much attention.

In filling materials the combination of tin and gold was commended as almost the ideal filling. Implantation was not considered as likely to prove a permanent operation; due to the absorption of the roots after a few months. It has not yet stood the test of time.

The practical results from the studies of the germ theory were found wanting; all has not been proved that has been claimed. In literature the lack of original matter in our periodical literature was deplored, the different journals being largely reprints one of the other; members of the profession were urged to contribute more freely from their stores of knowledge and experience.

In the discussion which followed Dr. Brophy said: We were still in want of a properly constructed instrument with which to prepare roots for the perfect adaptation of the crown to all irregularities and depressions. He thought the band should be so softened and annealed as to be burnished with all depressions. The root should also be thoroughly treated; the system of immediate filling now in vogue incurred great risks of acute pericementitis.

*Dr. Harlan*—Thought the paper conspicuous for what it had omitted especially as to many recent dental publications which were not even named. It was not the duty of the committee to discuss modes of practice but to report what was new.

*Dr: Noyes*—Thought the recent investigations in the germ theory had done much good in enabling us to do scientifically what we were before doing empirically. Our practice in cases of pus infection, inflamed pulps, pyorrhea alveolaris, etc., had been greatly modified through the influence of those investigations.

*Dr. Cushing*—Said that although the committee had evidently not read that paragraph of the constitution which defined their duties, still the report was a great improvement on any preceding report.

*Dr. Kitchen*—Thought that casting discredit on the report of Dr. Koch made last year which was a very valuable paper.

*Dr. Freeman*—Expected the report to be a *resume* of all new publications for the benefit of those who could not afford to purchase every thing new that came out.

*Dr. Sitherwood*—Thought it should be a review of dental literature.

*Dr. J. J. R. Patrick*—Considered it the duty of the committee to examine all new publications and point out all palpable errors. He thought the editors of the journals should exercise closer supervision and not publish every thing that was offered by patrons of the house that issued the journal.

*Dr. Cushing*—Defined the duty of the committee not to review but to tell what was durable and valuable.

The Committee on Dental Art and Inventions reported favorably on the Knapp blow pipe; Dunn's medical syringe; Dr. Wooley's root and canal dryer, with platinum broach; the S. S. White gold lining for rubber plates, and a variety of clamps, separators, matrices and other small appliances; also Dr. W. G. A. Bonwill's method of packing amalgam with bibulous paper.

*Dr. J. J. R. Patrick*, Belleville, read a paper entitled

DENTAL MORPHOLOGY AND THE ETIOLOGY OF IRREGULARITIES.

He said that the development of all life, whether animal or vegetable was from the simple cell, the fertilization of an ovule by the coalescence of the paternal and maternal principles. After amalgamation a change takes place; the cell becomes the germ; thence the embryo, fœtus, child, adult; the individuality begins with the primative cell. By segmentation other cells are developed, one surrounding the other until we have the epiblasts, the hylioblasts, and lastly the microblasts, each becoming granular nucleated spheres, specialized for function but all beginning in the same manner.

In the development of the teeth, much is still obscure. Taking the jaw of a young hog of six or eight months old, which is homologous with that of a child of six years, in studying the molars we find a molar in the saccular stage; each sac is connected with the gum by a fumete, an umbilicus in the truest sense of the term, of which the sac is the placenta, its connection with the parent stock presenting the same phenomena for nourishment dependent on the parent. As development progresses the funnicle becomes detached, and nutrient vessels for the formation of cancellated bone emerge from the alveolus and connect with the sac. Cementum has formed before the cusps unite. As the tooth progresses further, the funnicle becomes detached and a mutual interchange of circulation is accomplished; the plates of the alveolus part and allow the enamel crown to emerge. There is no absorption—not a particle. That is a term which explains nothing. When a thing is absorbed we want to know where it has gone; whether it is internal or external absorption. At the point of cleavage of the alveolus, as the crown emerges, the sac slips back to the neck, and as the roots approach completion the sac rounds over the roots and becomes the formative organ of cementum—known as Nasmyth's membrane, through which nutrient vessels penetrate and nourish the interior, as the synovial fluid filters through the membrane.

The teeth of the inferior maxillary are nourished by the inferior dental nerve and by the surrounding tissues.

Supernumerary teeth are not found in the lower jaw, except in pictures. They are frequent in the superior maxillary and are nourished by the surrounding tissues without any connection with the dental nerve though they develop enamel and dentine. Deciduous teeth are supplied by deciduous vessels and surrounded by deciduous bone and exuviated when useless.

*Dr. Angle*, Minneapolis, who had been appointed to open the discussion not being present, Dr. G. V. Black made the opening remarks. He said that though the main trend of the paper was correct he was obliged to disagree on some points, on which Dr. Patrick had peculiar views which surprised him somewhat. In the saccular stage the "umbilicus" would not be found so pronounced as in the illustrations used; neither had he ever seen any vessels in that cord.

The work of absorption is not a supposition; it can be clearly traced in bone, in connective tissue, but especially in hard tissues; it is removed by process of solution; cells grow in against it and cause it to dissolve, as the roots of the temporary teeth get away. As the tooth develops the surrounding bone is absorbed, disappearing like footprints in the snow; absorbed by organs brought into existence for that purpose. When nature has done with a thing it is thrown away, like a frog's tail.

As the crown is developed the outer layer of epithelium disappears; from impacted or belated teeth it disappears entirely, leaving the enamel in abnormal apposition with the tissues often causing great irritation. The membrane constitutes a support to the functioning cells. Supernumeraries in the lower jaw are rare but they are sometimes found. Has seen five lower incisors in one case, and four lower molars on either side, in another case. It was impossible to tell which were the abnormal teeth. He had recently seen an extra lateral incisor in a child of four years old, but they are very rare in the deciduous teeth. Teeth may be perfectly formed without nerve connection, but he had never seen any beyond the crypt stage. In one case a child was brought to full term without any trace of nervous system—the jaw was perfect and the teeth perfectly formed in the crypts.

No further discussion followed Dr. Black's remarks and Dr. Patrick concluded the subject by simply maintaining the position taken in the paper. He would not go back earlier than the microscope could demonstrate, mere belief and speculation retarding the progress of true science.

A communication was read from Dr. J. Rollo Knapp regretting his inability to be present with his promised paper on Dental Electrics, and his System of Gold Crown Work.

A committee was appointed to revise and reissue a pamphlet on Dental Hygiene for circulation among the people, in accordance with the suggestions of the President's address.

At the night session, Wednesday, Dr. C. C. Carroll, Meadville, Pa., gave a clinical lecture on his method of casting aluminum for dental purposess. The superior merit of aluminum for dental plates, because of its lightness, stiffness, strength, and freedom from oxidation has long been recognized; but its great shrinkage, and its disintegration in the fluids of the mouth have hitherto rendered its use impracticable. Dr. Carroll, after a long series of experiments, has succeeded in removing the impurities which caused the disintegration, and so alloying it as to practically overcome all shrinkage. By the use of a pneumatic crucible he overcomes its low specific gravity and forces the molten aluminum into every part of the matrix, thus producing very perfect casts.

For this work he takes an impression as for any other style of work, making a model of three parts plaster of Paris and one part marble dust. The wax base plate is made very thin, and very exact, as every line is reproduced in the casting. The plate can be cast and the teeth afterwards attached with pink rubber, or celluloid, or the metal may be cast directly to the teeth, plain teeth being used in preference, spacing them slightly ; gates and pockets are cut for the surplus and the casting made as soon as the matrix is dry. This method is used for crowns and bridges as well as for full or partial dentures.

At the close of this lecture, of which this is a mere outline, Dr. Carroll cast a full aluminum upper, in the matrix which had been drying out during the course of the lecture.

This was opened at the clinic the next morning and found to be perfect in every respect.

No discussion followed the lecture. Dr. L. Ottofy, Chicago, read a paper on

OPERATIVE DENTISTRY.

The scope of the subject is so large that he merely touched upon the many heads embraced in the title. He was opposed to the extravagantly large contour buildings sometimes indulged in, preferring porcelain settings or crowns, the less gold showing in the mouth the better. In crowning roots the band must be very exactly adapted to the root and cemented with oxyphosphate.

He had nothing new to report in filling materials or methods except the increasing use of tin and gold combined, which is most successfully used in such cavities as in the buccal surfaces of molars, etc. It has the great disadvantage of discoloration. This is also true of copper amalgam which is a great preservative of teeth of poor structure. The failure of amalgam fillings he attributed mainly to slovenly workmanship. Gutta percha he commended only for temporary fillings, and preferably the former. In the present craze for immediate root-filling he thought there was great lack of discrimination. There should be more efforts made to save the pulp alive by judicious capping with incorruptible, pliable substances. He commends chlor-percha and oxyphosphate. An abscess should be punctured, and treated from the outside. Matrices, separators, etc., should be used with discrimination.

In opening the discussion, Dr. Wassall spoke of the tendency to reduce the time of an operation with corresponding lack of thoroughness.

Would prefer a large contour operation for a young person. For crown operations prefers to the Logan or other crown, a plate tooth backed with gold, with a dowel, for front teeth. In bridgework there is a great field for usefulness where the case is suitable. He commended very highly Hood and Reynold's cylinders, as working like the Walrab gold, or S. S. White's velvet cylinders, but at much less cost. He thought the heavier foils were not used as much as they should be for hard surface work. Amalgam fillings should always be finished at a subsequent sitting that any defects or displacement might be remedied. The great secret in the successful use of the cements was to burnish it down thoroughly until it has a glossy vitrious surface like celluloid when pressed between tin foil. It will then withstand the action of saliva and of wear. The rubber dam should be left on for an hour after finishing. He considered the immediate filling of pulpless teeth with pulpless opening risking grave consequences even leading to caries of the bone. Would endeavor to save all pulps till maturity of the tooth—say eighteen or twenty years of age.

*Dr. Crouse*—Spoke of the proper treatment of new patients—study each case carefully—endeavor to ascertain the cause of previous failures — their knowledge of dental hygiene, etc. Take an easy operation at first to test them as patients. If necessary let them take a hand glass and with floss silk prove to them the lack of cleanliness; teach them that it is not vulgar to pick the teeth as young ladies are so often taught. Judge of the benefits to be conferred by your operations by the effects produced by your talk as seen at the next visit. If they won't keep their teeth clean after being taught how, no use to expend your vitality in fine contour operations. Considers Parr's separators superior to any thing yet invented; will separate where any thing can do it. If the teeth move easily they will get sore before the end of the operation; in that case it is better to contour first, and then separate for finishing. Prefers soft foil cylinders to the combination of tin and gold now in vogue—the old fashioned cylinders rolled on a broach. Does not like the velvet cylinders; prefers Kearsing's soft cohesive No. 10. William's gold is elegant, but Kearsing's takes less money. William's crystalloid gold spreads out well under the burnisher. As a system of bookkeeping: has a pad of diagrams on a page of which he enters the name and address of the patient, and marks all the cavities found on thorough examination; on this his assistant checks off the operations as made with the date and time occupied, to which he affixes the charge; the whole being daily transferred to the ledger. In this way there is no mixing up or forgetting.

At the afternoon session, Thursday, on motion of Dr. Harlan, a committee was appointed to memorialize Congress for removal of duties on dental materials and appliances.

A paper was read by Dr. W. B. Ames on

AMALGAMS.

He said that, though a champion of the plastics he was not a *New departurist.* He thought that their peculiar tenets had a ring of ambiguity; his reading would be just in proportion to the difficulty of holding decay in abeyance is amalgam the best material. Where tooth-structure is not in harmonious relation with the conditions necessary for gold, or when there is not accessibility, some other material than gold must be used. The use of tin-and-gold is limited to few locations, and where it can be used it is no better than amalgam. Gutta-percha and the cements are only valuable as adjuncts. Amalgam is easily manipulated, thoroughly plastic, and, when properly used, very valuable. Manipulation indicates the material in its composition. If heavily tinned we will have bulging; with copper we have but little change of form; a large proportion of silver gives a peculiar crepitation; gold in amalgam renders it dirty; copper controls changes of form, through imperfect amalgamation; particles of solid copper resisting change of form. Amalgamated platinum, though of unsightly color, and remaining indefinitely plastic by itself, imparts great toughness and edge strength; it is of an unctious, buttery consistance, and draws out like gum.

Palladium precipitate hastens the setting, and imparts toughness to other amalgams. But the main reliance is pure copper amalgam; its rapidity of setting can be regulated by manipulation, as the more it is rubbed and freed from mercury the more quickly it sets; the color and setting are both improved by washing with alcohol, if thoroughly washed it will have a steel-gray color, but this should not be done for very soft teeth.

Careful experiments had convinced him that the chemical effect of mercury in fillings is *nil*; neither does he anticipate any danger from the electro-chemical theory; electrolytic action would liberate nascent oxygen and sulphur; which would be beneficial in destroying micro-organisms. There might be acids, from recomposition often decomposition, but it would be on the surface of the metals and not on tooth substance.

*Dr. Taylor*, Streator, who was appointed to open the discussion of this paper, said that amalgam fillings had their limitation in three respects; their change in form; their color; their lack of edge-strength. That in their successful use more depends on method than material.

*Dr. Johnson*—Thought tin and gold superior to amalgam, and is inserted almost as readily.

*Dr. Garrett, Newkirk,* spoke strongly in favor of amalgams, for large compound fillings using a matrix cut from thin copper plate, and left in position until the patient returns the next day. With the matrix, amalgam can be packed in dry, without excess of material, consolidated and finished with the same care as for gold work, finishing with disks and strips, and polishing finely. He doubted if much copper amalgam was a healthy thing to have in the mouth.

The report of the Supervisor of Clinics occasioned a lengthy discussion of a variety of subjects, in which Drs. Crouse, Cushing, Richards, Reid, Pruyn, Koch, Sitherwood, Swain, Ottofy, Freeman, W. N. Morrison, Taylor, Moody, and others participated.

Drs. Sitherwood and Crouse were opposed to the use of cocaine or hypodermic injections for extracting; also to the use of matrices as a waste of time in adjusting, and being in the way when in position.

*Dr. W. N. Morrison*—Is also opposed to cocaine and much of the other medication now resorted to. Dr. Pruyn defended the use of cocaine, as having excellent results in the prevention of pain.

*Dr. Freeman*—Uses crystals of menthol in alcohol and a little chloroform as a local obtundent.

*D. J. D. Moody*—Mendota, read a paper on

THE CONSERVATIVE TREATMENT OF PULPS, and Dr. W. A. Johnston, Peoria, a paper on

INFLAMED PULPS.

The subjects of pulp-treatment, pulp-capping, and pulp-extirpation were discussed very fully. The conservation of the living pulp in young persons was urged. If the pulp cannot be saved, its painless removal can often be effected by the application of

Arsenic........................................1 part
Hydroch. cocaine..........................4 parts
Lanoline........................................5 parts

Drs. Crouse, Cushing, and some others favor a return to oxychloride of zinc for pulp capping. Dr. Crouse would coagulate the surface of the pulp with carbolic acid; Dr. Cushing covers it with a film of copal ether varnish.

*Dr. Cummings*—Gave the following formula for a sedative for exposed pulps:

4 per cent. vol. Hydrochlo. Cocaine......4 to 6 minims
Morphine..................................................$\frac{1}{2}$ grain made into a paste and dissolved in 20 minims of chloroform. This is to be applied to the pulp on one side of a piece of spunk, the other side being covered with copal ether varnish, and left in place from six to twenty-four hours, after which the tooth can be filled with amalgam or gold.

*Dr. Swain*—Expressed his gratification that some one had succeeded in capping pulps. He had never saved one, and had never seen one that had been saved!

*Dr. Crouse*—Asked if that was a question of integrity or of ability to detect exposure?

A paper from Dr. L. P. Haskell on

DIFFICULT CASES IN PROSTHETIC DENTISTRY, AND THEIR TREATMENT

was read by Dr. Harlan, in the absence of the author.

Failures are often due to the refusal of patients to be guided by the operator as to the removal of remaining teeth, roots, etc; also their refusal to wear a lower partial plate for missing molars —the occlusion of the inferior incisors displacing the upper denture. When the upper teeth are all gone except the six anterior teeth, and the bicuspids on one side, the latter will often have to be removed to equalize pressure; this will subserve the best interests of the patient. If two or three lower molars remain to meet a full upper set, they are best removed to equalize pressure; a full lower plate will be much more comfortable. Other similar cases were mentioned.

*Dr. Swain*—Opened the discussion of this paper. He insisted upon the importance of a thorough preliminary study of the mouth, as secondary only to a correct impression; it was a great mistake to suppose that plaster was always the best material for this. A very perfect impression may be obtained by first taking the impression in wax, and trimming that to proper size and shape to serve as an impression cup with very small quantity of plaster. A good rule for lower plates is to cut away until you are sure you have spoiled it and then cut away a little more, next to the cheeks! For partial lower plate we would use clasps even if sure they would destroy the teeth in a few years; those few years of comfortable ability to masticate would more than counterbalance the loss of the teeth. He agreed with the essayist that there were often cases in which the best interests of the patient demanded the extraction of sound teeth to make a success of the plate.

*Dr. Ames*—Thought the extraction of sound teeth utterly unjustifiable, except when there is an anterior space that cannot be filled with one tooth and yet will not admit two. Sound teeth should never be extracted because those on the other side are gone; an upper plate can always be constructed to meet any ordinary position of the lower teeth. Roots, if properly trimmed and filled afford a basis for firm pressure, and tend to steady the plate.

Friday morning Dr. J. Frank Marriner, Ottawa, read a valuable paper on

MAKING AND TEMPERING INSTRUMENTS.

This was one of the most practical papers read, and received close attention.

He said that great care must be taken not to overheat the steel, not to hammer before it reaches the right heat, and to turn it at each blow; it should not be forged too small, and should be finished by grinding and not filing. When the shape is just what is wanted—though still too large—with the blow pipe and the solid flame burner, it must be evenly rotated and heated to a cherry red, then suddenly plunged in cold water. The blade, neck and shank being now all of one temper, the blade must be forced into a bit of clean soft pine and the shank treated till the blue color passes up to the neck, and you will then have a spring temper at the neck with a clean cutting edge to the blade. It must now be ground on a stone kept constantly wet, until it is as thin and delicate as wanted, being held square and firm, and ground evenly. With practice and skill the shank may be made tough and strong, the neck firm and springy, and the blade keen and delicate—a thing of beauty and a joy—as long as it lasts.

The paper was discussed at some length by Drs. Cushing, Morrison, Wassall, Freeman and Swasey.

The subject was passed and Dr. J. J. R. Patrick read a paper entitled

### THE RATIONALE OF CONSTRUCTING AND ATTACHING ARTIFICIAL CROWNS TO NATURAL ROOTS OF TEETH.

The principles embodied in this paper were very clearly illustrated on the blackboard at every step, the illustrations forming an essential part of the lecture.

After an address from Dr. G. V. Black, embodying his resignation of the position of President of the State Board of Examiners, which was accepted with regrets, the committee appointed to witness Dr. Patrick's dissections of dental jaws, reported that the points made by Dr. Patrick in his paper on Dental Morphology had been fully sustained and shown clearly to the naked eye.

The hour for election of officers having arrived, Dr. Swain, seconded by Dr. Brophy, asked the unanimous voice of the society for the nomination of Dr. Geo. H. Cushing to the office of President, he being the only living member of the society who had been present at its organization, and who had attended every meeting since. It was therefore most fitting that he should preside over its quarter-centennial anniversary.

This nomination was greeted with warm applause, and Dr. Cushing was elected President with a unanimous rising vote.

Quincy was elected as the place of meeting. Dr. J. J. Jennelle, Cairo, was elected Vice President; Drs. Newkirk, Pritchett, and Ames were re-elected to the offices of Secretary, Treasurer and Librarian.

The Society then adjourned after the customary installation ceremonies.

---

CLINICS.

The morning sessions of Wednesday and Thursday were devoted to clinics.

*Dr. C. A. Kitchen*, Rockford, filled a large crown and anterior proximal cavity in an inferior molar, building with tin and finishing with gold. He used the new Belding Motor.

*Dr. J. W. Carmany*, Mt. Carroll, made a large gold contour filling in a right superior central incisor, using the electric mallet.

*Dr. F. L. Gilmer*, Quincy, inserted a telescopic platinum and gold crown, first adapting perfectly a very narrow platinum band, which can be more perfectly adjusted than the wider gold band which is slipped over it.

*Dr. K. B. Davis*, Springfield, filled a large anterior proximal cavity in a superior second bicuspid with tin and gold, laying a foundation of tin, followed by William's cylinder tin, finishing with No. 60 heavy foil.

*Dr. D. B. Freeman*, Chicago, filled with all gold large mesial and distal proximal cavities in the lower first and second molars, using a soft tin double-faced matrix. S. S. White's corrugated foil No. 4. was used in this filling.

*Dr. Brophy*, Chicago, filled a posterior proximal cavity in a right superior second bicuspid, using his continuous-band-matrix, filling with tin and gold to the cervical border. Dr. Brophy used the Detroit Company's motor.

*Dr. J. G. Reid*, Chicago, with tin and semi-cohesive gold, and using the Belding motor, filled an anterior proximal cavity in the superior first molar.

*Dr. E. D. Swain*, Chicago, filled an anterior proximal cavity in a right superior bicuspid, using William's cylinders annealed.

*Dr. Call* of Peoria, Dr. Patrick of Bellville, and Dr. I. S. Coyle of St. Louis, were present with methods for gold crowns. In the latter method the cap is stamped from coin gold and filled with 18 carat solder.

*Dr. I. Austin Dunn*, Chicago, demonstrated the value of his medicinal syringes for forcing medicaments through fistulous openings. In one case treated the superior first bicuspid had a chronic abscess of nine years standing. In another case, also a superior first bicuspid, the broach could not be passed through the apex, but the syringe forced peroxide of hydrogen freely through two fistulous tracts, one opening on the outside of the gum, and the other on the palatal surface. After discharging the pus, eugenol was passed through, followed by warm listerine, and then by bichloride of mercury. The root was then filled with gutta percha cones. Parson's hot-air-injector was used to dry out the canals.

*Dr. A. W. Harlan*—Treated a very bad case of Pyorrhea Alveolaris, all of the lower teeth, and a superior right bicuspid being involved. The teeth were loaded with calculus, several of them loose, with pus exuding from the margins of the gums. The case was probably of fifteen years standing. After the surgical removal of calculus and injection of peroxide of hydrogen, a ten per cent. solution of Recorcin was used, the final treatment being with iodine trichloride—the latest discovery in this line.

*Dr. C. C. Carroll*, Meadville, Pa., made a very perfect aluminum casting with his new method and apparatus, and instructed a large and interested class in his method.

*Dr. W. B. Ames*, Chicago, demonstrated the method of using copper amalgam by heating, rubbing, washing, squeezing, etc.

*Dr. J. J. R. Patrick*—Crowned a first right inferior bicuspid, and Dr. T. L. Gilmer crowned the left inferior bicuspid for the same patient.

*Dr. Chas. Pruyn*, Chicago, demonstrated the value of cocaine in extracting. In one case toxic results followed, which were promptly overcome by the proper antidotes.

One of the teeth—a left inferior molar—extracted by Dr. Pruyn, was replanted by Dr. W. N. Morrison, the root being filled with gutta percha and the crown cavity with amalgam.

*Dr. J. J. R. Patrick*—Exhibited his large working model and regulating appliances.

*Dr. W. N. Morrison*—Also exhibited his appliances for regulating, consisting of jack screws, platinum bands, springs, wedges, etc.

*Dr. W. H. Taggart*, Freeport, had a very convenient and economical apparatus for making corrundum points and disks from broken scraps of corrundum wheels and disks.

*Dr. Fuller*, St. Louis, had on exhibition a very compact and convenient wall cabinet for dental engine appliances.

Parson's electric warm air injector, saliva ejector, atomizer combined apparatus, attracted much attention and were used with satisfaction in some of the clinics. In this apparatus air is forced with a receiver, by means of an air pump, and a range of heat from 90° to 150° is obtained by means of a battery of two cells, which is transmitted through an insulated hand piece or electro-thermo-stat.

The current of air can also be utilized as a pneumatic plugger, or as a blow pipe.

The Belding Motor, which gave great satisfaction in the clinics, claims to be "the lightest, simplest, cheapest and most perfect motor in the world." It has the advantage of being attached directly to any engine without any special stand or impression arrangement.

The Detroit motor is offered wound for the Edison, Brush, and other circuits, and ranging in power up to one-quarter horse power.

There were on exhibition numerous clamps, separators, matrices, etc. Among the latter, Reid's Universal Matrix Appliance, for which the band is cut from "tagger's tin" to suit each case, a single "yoke and screw" being applicable in all cases, and costing no more than a single matrix of any of the different sets now on the market.

